# Atherosclerosis, inflammation and lipoprotein glomerulopathy in kidneys of apoE^-/-^/LDL^-/- ^double knockout mice

**DOI:** 10.1186/1471-2369-11-18

**Published:** 2010-08-20

**Authors:** Alexander C Langheinrich, Marian Kampschulte, Franziska Scheiter, Christian Dierkes, Philip Stieger, Rainer M Bohle, Wolfgang Weidner

**Affiliations:** 1 Department of Radiology, Justus-Liebig University Giessen, Giessen, Germany; 2 Department of Urology, Pediatric Urology and Andrology, Justus-Liebig University Giessen, Giessen, Germany; 3 Department of Pathology, Justus-Liebig University Giessen, Giessen, Germany; 4 Department of Pathology, Saarland University, Homburg, Germany; 5 Department of Cardiology, Justus-Liebig University Giessen, Giessen, Germany

## Abstract

**Background:**

The apoE^-/-^/LDL^-/- ^double knockout mice are bearing considerable structural homology to human atherosclerosis. We hypothesized, that advanced lesion formation in the renal artery is associated with kidney alterations in these mice.

**Methods:**

Kidneys from apoE^-/-^/LDL^-/- ^double knockout mice at the age of 80 weeks (n = 6) and C57/BL control mice (n = 5) were infused with Microfil, harvested and scanned with micro-CT (12 μm cubic voxels) and Nano-CT (900 nm cubic voxels). We quantitated the total vascular volume using micro-CT. Number and cross-sectional area (μm^2^) of glomeruli were measured using histology.

**Results:**

At the age of 80 weeks, the renal total vascular volume fraction decreased significantly (p < 0.001) compared to controls. Moreover, the renal artery showed advanced atherosclerotic lesions with adventitial Vasa vasorum neovascularization. Perivascular inflammation was present in kidneys of apoE^-/-^/LDL^-/- ^double knockout mice, predominantly involved are plasma cells and leucocytes. Glomeruli cross-sectional area (9959 ± 1083 μm^2^) and number (24.8 ± 4.5) increased in apoE^-/-^/LDL^-/- ^double knockout mice compared to controls (3533 ± 398 μm^2^; 17.6 ± 3, respectively), whereas 41% of the total number of glomeruli showed evidence for lipoprotein associated glomerulopathy (LPG). Moreover, immunohistochemistry demonstrated capillary aneurysms of the glomeruli filled with factor 8 containing emboli.

**Conclusion:**

The reduced intra-renal total vascular volume is associated with systemic atherosclerosis and glomeruli alterations in the apoE^-/-^/LDL^-/- ^double knockout mouse model.

## Background

Chronic inflammation of large blood vessels, as seen in atherosclerosis, is associated with kidney dysfunction [1] and lipoprotein disorders [2]. In broad outline, the relation of systemic atherosclerosis and kidney function has been documented [3,4] and the magnitude of renal dysfunction is related to cardiovascular risk factors [5].

In the past, animal models of acute and chronic kidney dysfunction have been widely used mimicking human disease [6-11]. Previously, it has been demonstrated that the apoE^-/- ^mouse spontaneously develops lipoprotein glomerulopathy (LPG) at the age of 86 weeks [12] and variations in the composition in dilated glomeruli lumens of apoE^-/- ^mice compared to the apoE-Sendai mouse have been demonstrated.

This study is a continuation of our characterization of advanced atherosclerotic lesions in aortas of male apoE^-/-^/LDL^-/- ^double knockout mice [13]. In that study we reported that the spatial location and magnitude of Vasa vasorum density and adventitial inflammation were strongly correlated in advanced atherosclerotic lesions and identified as an independent correlate to different categories of advanced lesion types. Inflammation in this mouse model has been shown in various organs, including the lung, heart and aorta [14].

The present study was designed to test the potential for quantitative imaging the renal vasculature as a marker of kidney function in this mouse model of advanced atherosclerosis. For analysis of the renal vascular volume we used high-resolution micro-CT as well as histology to localize, identify and quantitate regions of plaque formation and inflammation and its consequences as far as kidney alterations are concerned.

## Methods

### Experimental design

Experiments were performed according to the "German Animal-Protection Law" (1993). Approval of the institutional animal care and use committee was obtained before the start of this study.

Male apoE^-/-^/LDL^-/- ^double knockout mice on a standard diet were (Charles Rivers Wiga, Sulzbach, Germany) euthanized after 80 (n = 6) weeks with a fatal dose of inhalative trichlormethane. Age-matched male C57/BL mice (n = 5) on a standard diet served as controls. The left ventricle was cannulated and infused with heparinized saline (10 ml of 0.9% sodium chloride with 1000 IE Heparin) until the venous effluent was free of blood. A lead-containing radiopaque (Microfil MV-122, Flow Tech, Carver, MA, USA) at a nominal pressure of 100 mmHg was infused. After polymerization of the compound, the kidneys were gathered in toto and immediately immersed in 4% neutral buffered formalin.

### Micro- and Nano-CT Imaging

First, the kidneys were scanned in toto by a Micro-CT system described recently [13]. The resulting 3D images were displayed using image analysis software (Analyze^® ^6.0; Biomedical Imaging Resource, Mayo Clinic, Rochester, MN). For this study, the Micro-CT scanner was configured so that the side dimension of the cubic voxels was 12 μm (8-bit grayscale). Next, samples were re-scanned at 900 nm voxel size at 12-bit grayscale for more detailed analysis of the microvascular anatomy of the kidney using a nano-computed tomograph (Nano-CT_2011), manufactured and developed by SkyScan^® ^(Kontich, Belgium). The microfocus X-ray source is a pumped type source (open type x-ray source) with a LaB6 cathode. The electron beam is focused by two electromagnetic lenses onto the surface of an x-ray target. The x-ray target (Au) contains a thin tungsten film plated on the surface of the beryllium window producing x-ray emission reaching a minimum spot size of < 400 nm. At this small spot size, small-angle scattering enhances object details down to 150 nm isotropic voxels size. The X-ray detector consists of a 12-bit digital, water-cooled CCD high-resolution (1280 × 1024 pixel) camera with fibre optic 3.7:1 coupling to an X-ray scintillator and digital frame-grabber. In our experimental setting, samples were positioned on a computer controlled rotation stage and scanned 180° around the vertical axis in rotation steps of 0.25 degrees at 40 kVp.

### Histology

All kidneys from apoE^-/-^/LDL^-/- ^double knockout mice (n = 12) and C57/BL (n = 10) were embedded in paraffin wax and cut into cross-sections of 6 μm with a microtome. Serial sections were mounted on a microscope slide and stained with hematoxylin & eosin and PAS (Periodic acid-Schiff stain).

For immunohistochemistry, paraffin-embedded tissue sections (5-micron) were stained after 1% pronase digestion using the DAKO TechMate system (Glostrup, DK) with rabbit polyclonal antibodies against factor VIII-related antigen (DAKO, A0082) diluted 1:12.000 in antibody diluent (DAKO, S2022). Mouse-anti-rabbit antibodies (DAKO, M0373, 1:200) and link antibodies, APAAP complex and chromogenes from the ChemMate kit (DAKO, K5000) were used followed by counterstaining with hematoxylin (DAKO, S2020, 1:2). Total number of glomeruli, cross-sectional surface area (μm^2^) and the capillary diameter (μm) within the glomeruli were measured in all samples. Cross-sections were digitalized and analyzed by an experienced pathologist (R. M. B.).

### Statistical Analysis

Statistical analysis was performed using JMP 6.0 (SAS Institute, Cary, NC, USA). All data in the text and figures are presented as mean ± SEM. Vascular volume fraction, number and size of glomeruli were analyzed using unpaired t test and one-way ANOVA. A value of p < 0.05 was considered significant in all analyses.

## Results

### Atherosclerotic Lesions and Inflammation

**Figure 1:**
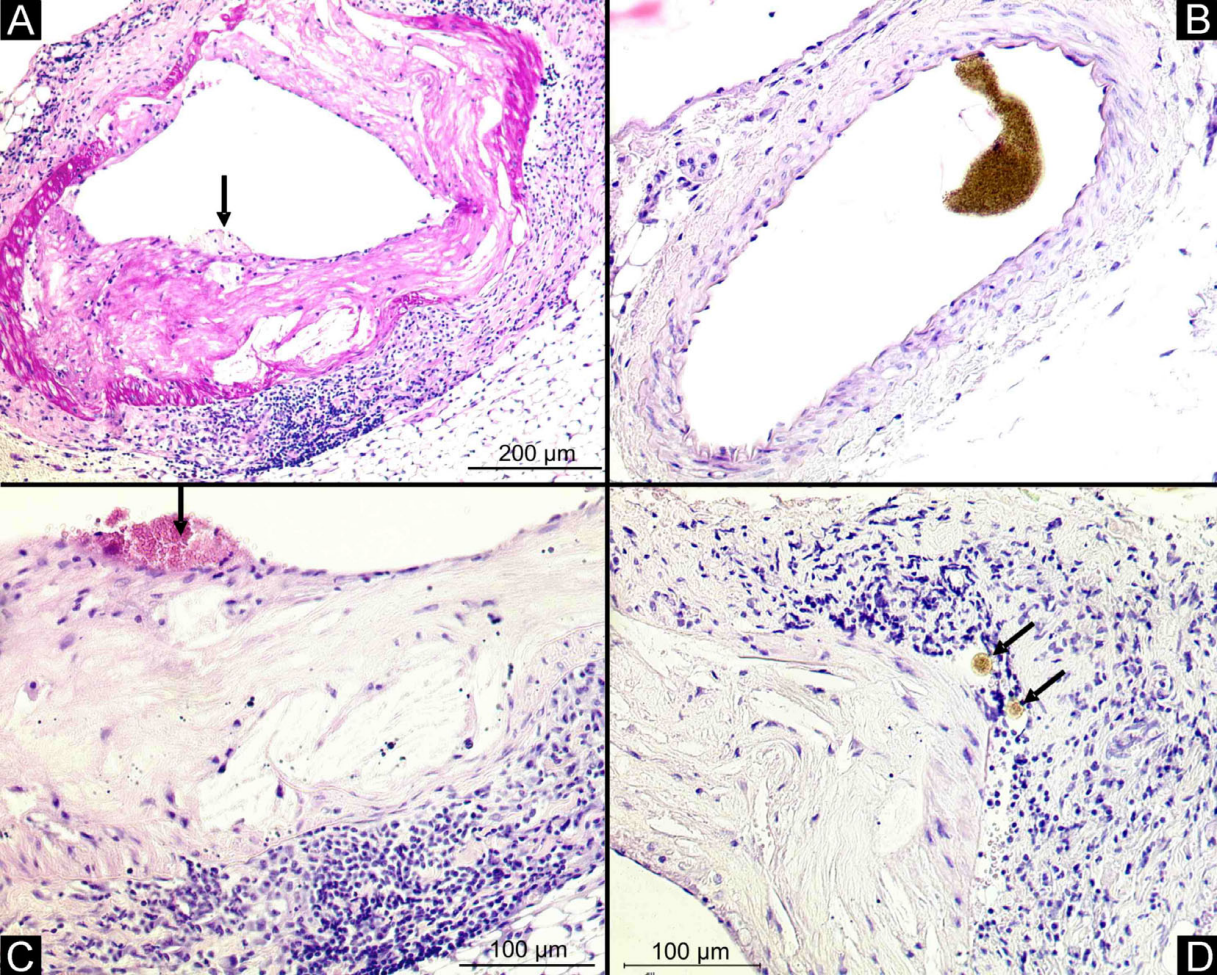
**Renal artery**. Histology demonstrates concentric atherosclerotic plaques in the renal artery of the apoE^-/-^/LDL^-/- ^double knockout mouse at the age of 80 weeks (A, magnification × 50). Control animals showed no atherosclerotic lesions in the renal artery (B, magnification × 50). In high-power fields, plaque rupture (C, black arrow, magnification × 100) and contrast enhanced Vasa vasorum (D, black arrow, magnification × 100) in the adventitia with large clusters of inflammatory cells occur.

As determined by micro-CT and histology, the renal artery from apoE^-/-^/LDL^-/- ^double knockout mice at the age of 80 weeks showed advanced atherosclerotic plaques with adventitial Vasa vasorum neovascularization and adventitial inflammation (Figure 1). The intrarenal vasculature with a diameter less than 400 μm did not show any atherosclerotic lesions. In contrast, the renal artery of C57/BL controls showed no atherosclerotic lesions and no perivascular inflammation.

### Renal Inflammation

**Figure 2:**
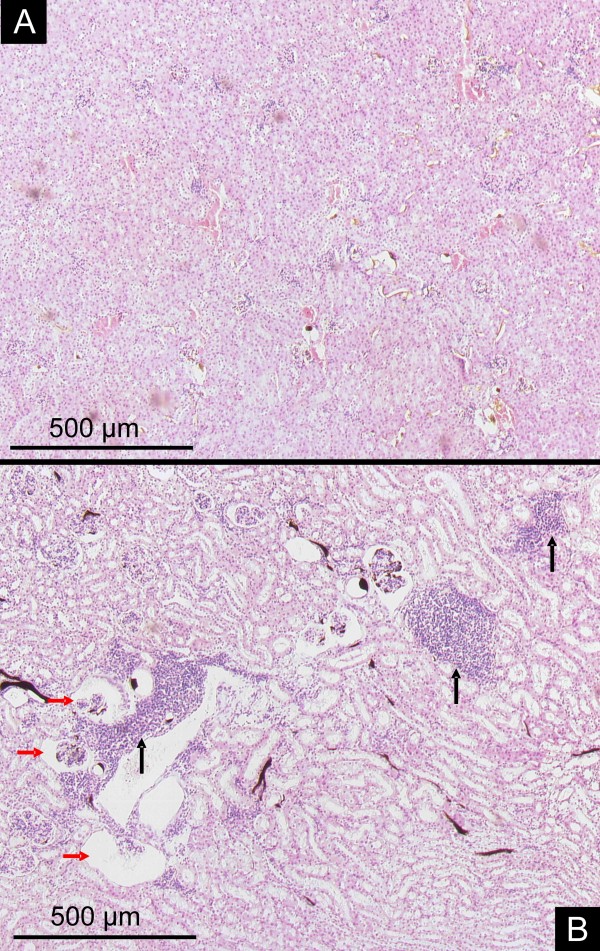
**Parenchymal alterations**. Control animals with normal kidney architecture and regular glomeruli (HE stain, magnification × 25). In contrast, kidneys of apoE^-/- ^/LDL^-/- ^double knockout mice demonstrate lipoprotein glomerulopathy (B, red arrows) and perivascular clusters of inflammatory cells (B, black arrows, magnification × 25).

**Figure 3:**
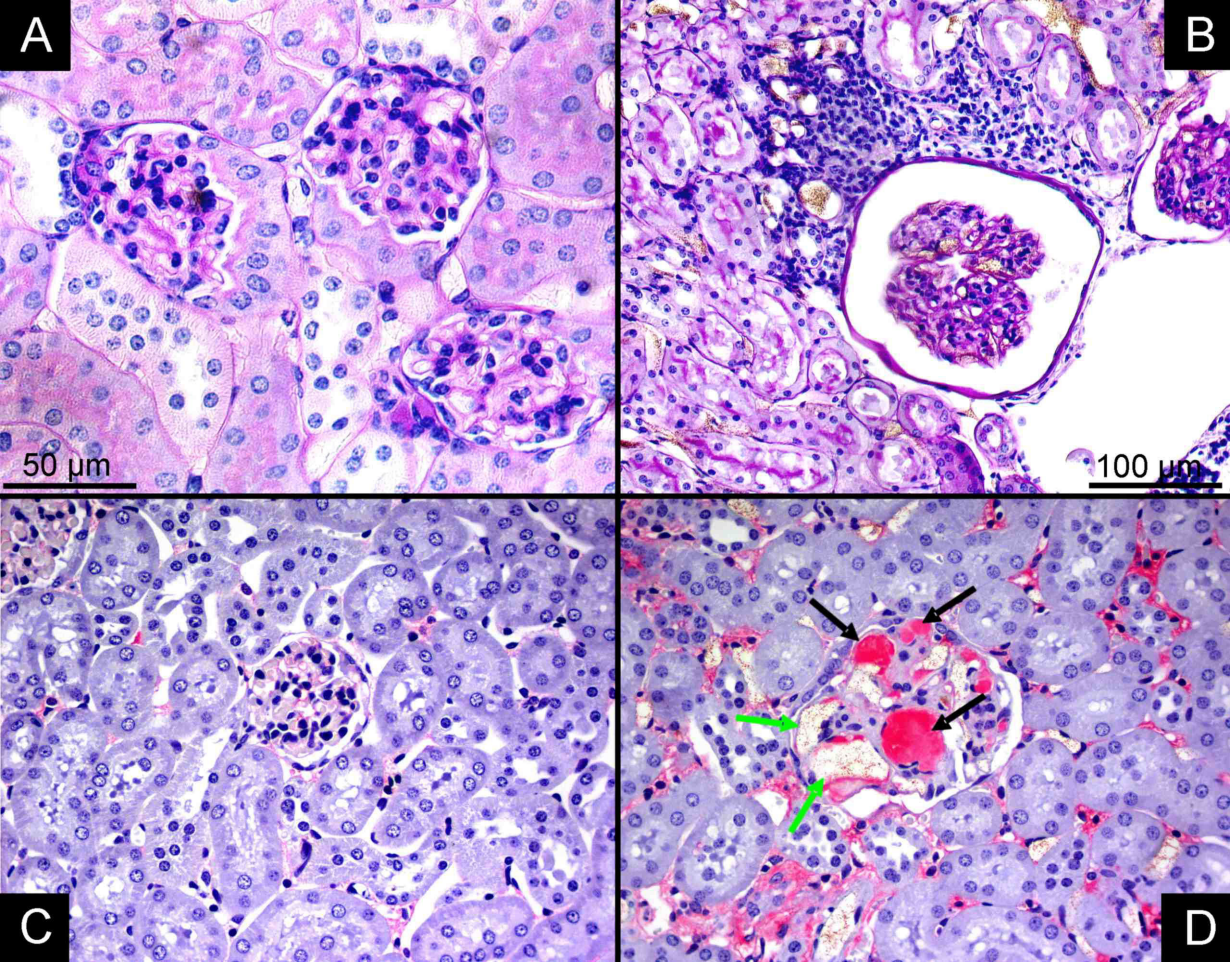
**Immunohistochemestry**. Glomeruli from controls (A, PAS staining; C, anti von-Willebrand Factor) and apoE^-/-^/LDL^-/- ^double knockout mice (B, PAS staining; D, anti von-Willebrand Factor). Perivascular inflammation (B) and capillary aneurysms of the glomeruli (D, green arrow) filled with factor 8 containing emboli (D, black arrow) are demonstrated. (A, C, D, magnification × 200; B, magnification × 100)

Vessels with a diameter between 150 to 300 μm were surrounded by large clusters of inflammatory cells (Figure 2). Histology demonstrated mixed inflammation, predominantly involved are leucocytes and plasma cells. Immunohistochemistry showed capillary aneurysms of the glomeruli filled with factor 8 containing emboli (Figure 3).

### Quantitative Measurements

**Figure 4:**
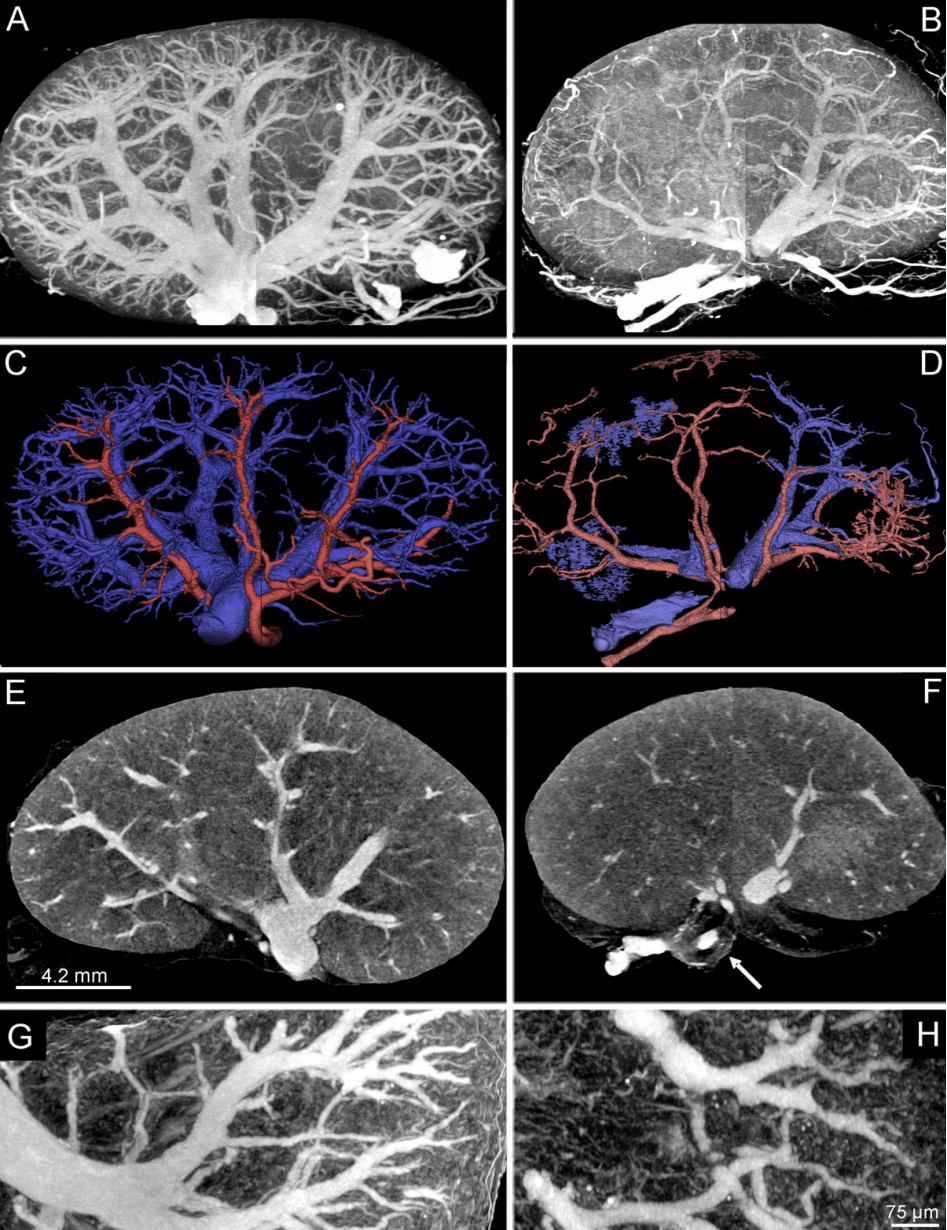
**Quantitative 3D micro-CT imaging**. Micro-CT imaging (maximum intensity projection, A, B, G, H; single-slice, E, F) of controls (A, C, E, G) and apoE^-/-^/LDL^-/- ^double knockout mice (B, D, F, H). Reduced intrarenal vascular volume is present in apoE^-/-^/LDL^-/- ^double knockout mice at the age of 80 weeks (B, D) compared to controls (A, C). Volume-rendering from controls (C) and double-knockout mice (D) demonstrate the reduced vascular perfusion territories of the intra-renal arterial (red) and venous (blue) vasculature. Nano-CT imaging demonstrates irregular vascular branching with luminal enchroachments in apoE^-/-^/LDL^-/- ^double knockout mice (F) compared to controls (E).

**Figure 5:**
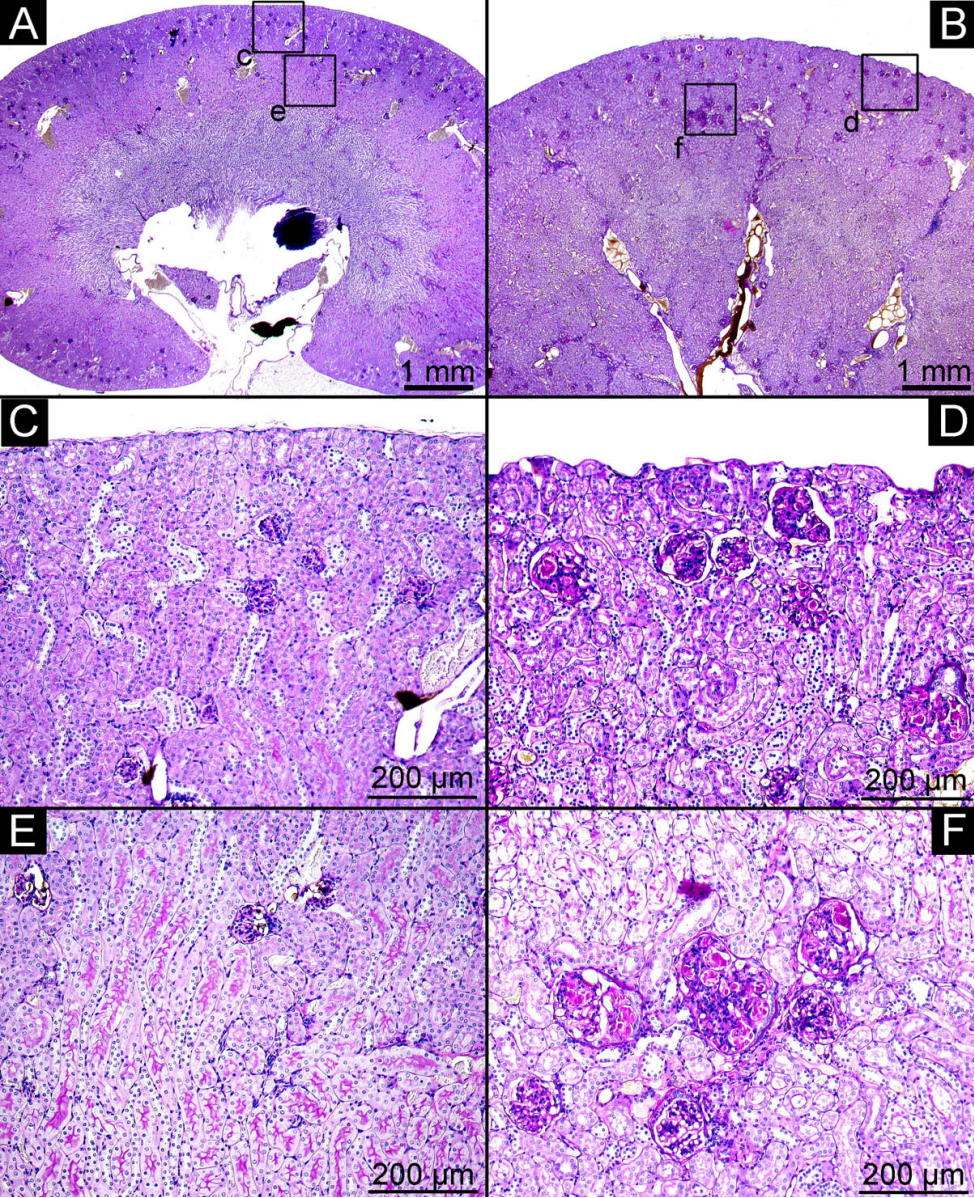
**Histology**. Control animals (A, C, E) with normal kidney architecture and regular glomeruli from the cortex (C) and the cortico-medullar junction (E) (PAS stain, A, magnification, ×12.5, C, E, PAS stain, magnification × 100). In contrast, kidneys of apoE^-/-^/LDL^-/- ^double knockout mice (B, D, F) demonstrate lipoprotein glomerulopathy (D, F) from the cortex (D, PAS stain, magnification × 100) and the cortic-medullar junction (F, PAS stain, magnification × 100).

Quantitative measurements are summarized in Table 1. Micro- and Nano-CT imaging demonstrated a significant reduction in the total vascular volume in apoE^-/-^/LDL^-/- ^double knockout mice at the age of 80 weeks compared to controls (3.2 ± 0.3 mm^3 ^vs. 4.3 ± 0.4 mm^2^, respectively; p < 0.001, Figure 4). Separation of the intra-renal arterial and venous vasculature demonstrates a significant decrease of the venous vascular volume fraction in apoE-LDL double knockout mice compared to controls (Table 1). As shown in Figure 4, the renal vasculature demonstrated an inhomogeneous branching pattern with luminal encroachments. The glomeruli cross-sectional area (9959 ± 1083 μm^2^) and total number/cross-section (24.8 ± 4.5) increased in apoE^-/-^/LDL^-/- ^double knockout mice compared to controls (3533 ± 398 μm^2^, 17.6 ± 3, respectively), whereas 41% of the total number of glomeruli showed evidence for lipoprotein associated glomerulopathy (Figure 5). Dilated glomerular capillaries were found in 85% of all glomeruli in apoE^-/-^/LDL^-/- ^double knockout mice compared to controls (22.3 ± 3.9 vs. 8.2 ± 2.3 μm).

**Table 1:** Quantitative micro-CT measurements

	C57/BL mouse (Controls)	**ApoE**^**-/-**^**/LDL**^**-/- **^**double knockout mouse**
**Total Vascular Volume Fraction (mm**^**3**^**)**	4.3 ± 0.4	3.2 ± 0.3*
**Venous Vascular Volume Fraction (mm**^**3**^**)**	3.9 ± 0.3	2.9 ± 0.4*
**Arterial Vascular Volume Fraction (mm**^**3**^**)**	0.4 ± 0.1	0.3 ± 0.1
**Maximum Kidney Dimension (mm)**	10.6 ± 1.7	11.5 ± 1.9
**Number of Glomeruli (per cross-section)**	17 ± 3	24 ± 5*
**Number of Diseased Glomeruli (per cross-section**	0	11 ± 3*
**Glomeruli cross-sectional surface area (μm**^**2**^**)**	3533 ± 358	9959 ± 658*
**Diameter Glomerular Capillaries (μm)**	8.2 ± 2.3	22.3 ± 3.9*

A significant decrease in the total vascular volume fraction and a significant increase in the total number and area of glomeruli is present in apoE^-/-^/LDL^-/- ^double knockout mice compared to controls (*p < 0.001). The decrease of the total vascular volume fraction is due to the decrease in the venous vascular volume fraction (p < 0.01). No significant difference in the arterial vascular volume fraction was found between the groups (p < 0.4).

## Discussion

The present study demonstrates advanced atherosclerotic lesions in the renal artery and peri-vascular inflammation in kidneys of apoE^-/-^/LDL^-/- ^double knockout mice at the age of 80 weeks. Moreover, capillary aneurysms with factor 8 containing emboli are present in apoE^-/-^/LDL^-/- ^double knockout mice at the age of 80 weeks.

Atherosclerosis represents one of the major causes of death in the western world and is associated with chronic kidney disease [15]. There is evidence that progressive deterioration of renal function in chronic kidney disease may also lead to dyslipidemia, thereby inducing the production of free-radicals and proinflammatory factors leading to endothelial cell dysfunction. This circle may facilitate and promote atherogenesis [1,11,16-18].

The apoE^-/-^/LDL^-/- ^double knockout mouse develops advanced atherosclerotic lesions with high similarity to human disease [13]. This murine model that lack the gene encoding apoE and LDL receptor knockout develop spontaneous hypercholesterolemia/hyperlipoproteinemia [19]. In the present study, the renal artery contains atherosclerotic lesions at the age of 80 weeks, similar to those found in the aorta, as described previously [13]. Plaque characterization showed advanced lesions, adventitial inflammation and Vasa vasorum neovascularization. No atherosclerotic lesions were found in the intrarenal arteries with a diameter less than 0.4 mm. Intrarenal arteries with a diameter between 150 to 300 μm were surrounded by large clusters of inflammatory cells. These affected vessels were free of any atherosclerotic changes. Previously, same results were obtained for the intrapulmonary arteries in the same mouse model of atherosclerosis [14].

Necrotising media arteriitis with large clusters of inflammatory cells in the adventitia have been reported in renal arteries due to systemic hypertension [20,21]. Hence, one can speculate, that peri-vascular inflammation is not necessarily associated with lesion formation per se, but with systemic atherogenesis and related to the vessel diameter.

Coexistence of hypercholesterolemia and hypertension increased Vasa vasorum density in rats [22], this is of particular interest, because our animals showed severe atherosclerotic lesions in the renal artery and therefore, we can not exclude hypertension due to hemodynamic relevant renal artery stenosis. We did not measure the intra-arterial systemic blood pressure, but, the left ventricle our apoE^-/-^/LDL^-/- ^double knockout mice showed an increase in wall thickness compared to controls as demonstrated previously [13].

Variants of apolipoprotein E have been linked to lipoprotein glomerulopathy (LPG), a glomerular disease characterized by the deposition of lipoproteins in glomerular capillaries [23,24]. Studies showed that cholesterol-feeding to various experimental animals induced the development of glomerular injury. Treatment of hyperlipidemic animals with lipid lowering drugs prevented the development of glomerulopathy. The apoE^-/- ^mouse spontaneously develops LPG at the age of 86 weeks [12] and variations in the composition in dilated glomeruli lumens of apoE^-/- ^mice compared to the apoE-Sendai mouse have been demonstrated. In this study [12], the link of peri-vascular and/or interstitial inflammation and/or advanced atherosclerotic lesion formation in the renal artery has not been described. Consequently, a pleiotropic effect including ageing, diabetes, advanced, systemic atherosclerosis with associated inflammation and hypercholesterolemia might be involved in LPG.

## Conclusions

Systemic atherosclerosis, Vasa vasorum neovascularization and parenchymal inflammation are seemingly inseparably linked, perhaps triggered and perpetuated by inflammatory reactions within the vascular wall. In addition to this mouse model providing a convenient method of following the progression of the systemic atherosclerotic process, it provides potential for a thorough and rapid evaluation of methods to arrest or reverse kidney alterations. As quantitative information of the vasculature can be obtained using high-resolution micro-CT, imaging and monitoring of inflammatory reactions during the onset, during the course and e.g. after anti-inflammatory therapy of atherosclerosis can be qualitatively and quantitatively performed.

## Competing interests

The authors declare that they have no competing interests.

## Authors' contributions

ACL, MK, FS carried out the micro-CT studies and drafted the manuscript. Histopathology was performed by RMB and CD. PS and WW participated in its design and coordination and helped to draft the manuscript. All authors read and approved the final manuscript.

## Pre-publication history

The pre-publication history for this paper can be accessed here:

http://www.biomedcentral.com/1471-2369/11/18/prepub
